# Critical bioethics: a new paradigm for the era of techno-feudalism

**DOI:** 10.1007/s11019-026-10360-4

**Published:** 2026-05-11

**Authors:** Giovanni Rubeis

**Affiliations:** Division of Health Care Ethics, University of Graz, Heinrichstr. 78, 8010 Graz, Austria

**Keywords:** Big tech, Critical theory, Health equity, Power asymmetries, Racism, Right-wing extremism

## Abstract

The rise of right-wing political movements supported by key players from big tech is transforming Western societies. This so-called techno-feudalism also implies challenges for bioethics. Racist agendas, intentional misinformation on health topics such as vaccinations, banned words and research topics in public institutions, and the massive funding of questionable biomedical research projects undermine health equity, patient safety, and autonomy. This new sociopolitical situation exacerbates a long-standing issue in healthcare that bioethics hitherto mostly failed to address adequately: societal power asymmetries that shape the roles and relationships of actors in healthcare and biomedical research. Although attempts have been made to reflect upon this issue, e.g. in feminist or postcolonial bioethics, there is no coherent bioethical approach that fundamentally focuses on power asymmetries as a lens of bioethical inquiry. In this article, I therefore introduce critical bioethics, an approach that takes epistemic lenses from critical theory, especially the so-called Frankfurt School. These epistemic lenses—totality and embeddedness, instrumental reason, dialectics and emancipation—allow us to uncover the societal causes for ethical issues in healthcare and biomedical research. Based on this methodological foundation, critical bioethics addresses health inequity as a result of power asymmetries by understanding individuals as fundamentally embedded in a concrete socioeconomic context. Through the lens of instrumental reason, it addresses the connection between disruptive technological innovations and economic interests in terms of commodification and solutionism. By using dialectics as a method to uncover contradictions in the way bioethics understands its principles, it provides an emancipatory perspective for normativity that separates it from affirmative forms of bioethical thinking that simply reproduce the suppressive status quo.

## Why critical bioethics?

The rise of right-wing populism and outright fascism, mostly fueled by libertarian economic ideologies and funded by the financial elites, is transforming Western societies and political orders (Geiselberg 2017; Milonas 2024). The second presidency of Donald J. Trump and the massive role Elon Musk and his tech-empire plays in this is just one of many examples of how the symbiosis of big tech and ultra-right political movements works. Some scholars speak of neo-feudalism (Dean 2025; Kotkin 2020) or techno-feudalism (Varoufakis 2023), while others see the rise of oligarchies (Zuboff 2022). One essential feature of this new paradigm is the control over crucial digital technologies, such as social media and health technologies. The power agglomeration in the hands of a few actors, which can go beyond any democratic control, is unprecedented and allows them to disrupt the social order of Western countries that existed since 1945. Key players in digital technologies often side with the ultra-right political forces and in part actively support them. At the same time, these actors control key technologies in data collection and analysis, especially machine learning algorithms, cloud solutions, and communication technologies. This process affects several domains of bioethics: Racist agendas as well as intentional misinformation on health topics such as vaccinations undermine health equity, patient safety, and autonomy (Southwell et al. 2023; Zimmerman 2024). Biomedical research is affected by political agendas and the growing influence of financial actors, which threaten scientific freedom. Companies like Alphabet, Meta, and Neuralink invest hundreds of millions of dollars in biomedical, especially neurological research and drug discovery (Schuhmacher et al. 2023; Webster 2023). Some authors speak of “datafeudalism” (Saura Garcia 2024) or “biosupremacy” (Marks 2020).

This social and political shift exacerbates ethical issues in contemporary healthcare, which can be reduced to a common denominator: societal power asymmetries that cause inequality and harm health equity. This has long been discussed as a major issue in bioethics with countless articles pointing out the relevance of social determinants of health and their impact on health equity as well as quality of care. Structural inequalities, i.e. unjustified differences in access to healthcare, power relations, as well as distribution and availability of resources have been widely discussed (Egede et al. 2024). It has been shown that these various forms of inequities cause adverse health outcomes for specific social groups or individuals. Social determinants of health have become a major focus not only in bioethical debates, but also in clinical practice.

And yet, bioethical inquiry has so far reacted to these phenomena in its traditional fashion, i.e. dealing with each of these issues separately. Some scholars have addressed either structural racism (Yearby 2021), gender inequality (Carpenter and Jordens 2022), or colonialism (Pratt and de Vries 2023) for example. Others have pointed out that bioethics needs to consider the societal context of healthcare practices and embrace social sciences for this purpose (Hedgecoe 2004). However, there is no coherent approach that focuses on the underlying cause of all of these outcomes, namely the power asymmetries inherent to a concrete socio-political system, in a methodological sense. It seems that despite occasional efforts, bioethical inquiry shies away from a conceptual approach that systematically uses social power asymmetries as an epistemic instrument for analyzing ethical issues in healthcare.

Recent political developments are about to exacerbate this situation. The new political constellation does not only ignore health disparities and structural discrimination, but actively propagates racism, gender inequality, and misinformation as well as defunding of public health and biomedical research that does not fit their right-wing agenda or economic goals. Hence, we are witnessing a transformation process, a social disruption that also affects many areas of bioethical inquiry, such as clinical medicine, public health, and biomedical research. The main challenges for bioethics resulting from this disruption are (1) exacerbated inequity and inequality as a result of aggravated power asymmetries inherent to societies in late capitalism, (2) technological innovations, especially digital technologies, that disrupt social practices and relationships and imply a concentration of power and wealth in the hands of a few, (3) the political suppression of minority groups and all who disagree with the techno-feudalist agenda. In order to respond to these challenges, bioethics has to develop a new approach of normativity. After all, bioethics is a branch of normative ethics and thus cannot simply ignore the need to take a stand in the face of social injustice and suppression. Being impassive in this regard means to be affirmative of the techno-feudalist paradigm.

Contemporary bioethics is largely unprepared for dealing with the reverberations of this particular sociopolitical situation because it lacks the necessary means to conceptualize and analyze its effects adequately. What is especially missing are tools that allow us to broaden the view of bioethics beyond analyzing isolated problems or conflicts like the patient-doctor dyad or protecting the autonomy of human research subjects. In other words, we need epistemic lenses that enable us to contextualize the analysis of bioethical issues with the broader social disruption that currently occurs. The good news is that these epistemic lenses already exist and simply have to be adapted to the requirements of bioethical research and analysis. I argue that critical theory provides these epistemic lenses. Hence, I aim to introduce *critical bioethics*, an approach that incorporates elements from critical theory and makes them usable for a normative bioethical analysis. I have discussed some preliminary thoughts on critical bioethics elsewhere (Rubeis 2024) which I aim to develop in full in this article.

In the following, I outline my approach, starting with some remarks on theory and theorizing in bioethics. Following this, I provide a short overview on the debate on what has hitherto been discussed under the label of critical bioethics. I argue that this discussion, although yielding some important results, misses the point of what “critical” ought to mean. I then discuss critical theory as a new perspective for bioethics, focusing on several epistemic lenses this approach can provide. I argue that these epistemic lenses allow bioethics to address the disruption of healthcare and biomedical research caused by the aforementioned sociopolitical transformation process.

## What bioethics?

As I pointed out above, bioethics in its current state is unable to deal with contemporary societal challenges. Similar observations have been made throughout the last three decades and the proposed solution has mostly been to embrace social sciences and empirical methods (Borry et al. 2005, Hoffmaster 1992, Muschenga 2005). Many scholars argued that bioethics needs an empirical approach in order to better understand the social forces that shape healthcare and biomedical research. This was mostly understood as a counter-movement against a theory-laden bioethics that is more concerned with discerning and weighing abstract principles and values than the real-world factors relevant to clinical decision-making or evaluating biomedical research. There has always been a strong tendency in bioethics to resent moral theory and philosophical theorizing (Banja 2022; Beauchamp 2004; Dunn and Sheehan 2024; Sherwin 1999; Toulmin 1981). This theory-phobic stance is still alive, although there have been advocates of theory in bioethics as well (Blumenthal-Barby et al. 2022; DeGrazia 2022; Holm 2024). Only recently, Holm (2024) could show that even those positions that claim to be purely empirical or explicitly refuse any theoretical foundations are very much reliant on some form of moral theory. It seems rather odd that a scientific discipline would be so fiercely opposed to theory, given the fact that theorizing is an essential component of any scientific endeavor. It is especially hard to grasp how we should deal with the contemporary situation where paradigms shift and whole societies change without analyzing and revising our basic concepts.

This theory-phobic attitude mostly stems from two fallacies: the reduction of bioethics to clinical ethics, i.e. decision-making in singular, presumably isolated clinical situations and a false dichotomy between theory and practice. Both fallacies are connected. When bioethics is simply defined in terms of providing the foundations for clinical decision-making, it becomes questionable how moral theory can be applied here. This is especially the case when supposing a mechanistic understanding of applied ethics, i.e. a top-down deductive application of universal, abstract principles.

A more enlightened view of theory and theorizing debunks this mechanistic interpretation and defines theory as a reflection on and analysis of the basic setting in which bioethical inquiry takes place as well as of basic concepts and principles. Even if we define clinical decision-making as a crucial task of bioethics, we have to concede that theory is necessary. How can we make sound and just decisions when we do not reflect on the determinants and social practices that shape the very situation in which a decision has to be made? The patient-doctor-dyad and clinical decision-making do not exist in a vacuum isolated from social structures, determinants, practices, and processes of social change. In fact, these factors shape the setting in which a decision is to be made, it shapes the actors and their options. Sure, embracing empirical methods is one way to account for these factors. However, all empirical data needs interpretation and analysis based on a sound conceptualization. What is more, it requires sharp epistemic lenses, i.e. concepts that help us to focus on the right empirical aspects and frame them adequately. In other words, we need theorizing in terms of a critical reflection on basic concepts and principles in bioethics.

## What criticism?

“Critical” can have at least three meanings in the context of bioethics. One meaning of “critical” refers to clarifying and challenging fundamental premises and assumptions (Garrett 2015). Critical thinking in this understanding means an evaluation of concepts, positions, and arguments that focuses on clarity, consistency, fairness, evidence, and sound reasoning as categories (Árnason 2015). The aim is to test whether assumptions and arguments are sound and valid and whether conclusions derived from them are correct or acceptable (Holm 2024). This interpretation of “critical bioethics”, which I would call *analytical criticism*, mainly focuses on applying methods and concepts from analytical philosophy, which implies mostly disregarding continental philosophy (Árnason 2015). The result is a discourse that centers around preventing harms and individual preferences, as well as analyzing value rationality and how to protect moral values or how to weigh them against each other (Engelhardt 2011; Garrett 2015). This can be considered as the mainstream in bioethics.

The second meaning of criticism has been referred to as *hermeneutical* (Árnason 2015) or *Socratic* (Dawson 2010) *critique*. It challenges established ways of thinking in bioethics as well as its dogmas like the emphasis on autonomy, voluntary contractual obligations, and laws and regulationqs (Dawson 2010). In addition, Hermeneutical or Socratic approaches aim to broaden the perspective of bioethics. Dawson (2010) for example suggests to include a public health view on bioethical problems in order to introduce concepts like equity, reciprocity, or solidarity for better framing bioethical problems and in general acknowledge social relationships as crucial factors.

The third meaning refers to sociological and anthropological approaches that analyze bioethics as a discipline and social practice (Hoeyer 2006; Turner 2009). This *social science critique of bioethics* (Hedgecoe 2004) criticizes the understanding of bioethics as applied ethics and advocating an empirical approach based on social sciences. Some of these approaches attempt a fundamental criticism of bioethics, i.e. either point out that bioethical concepts lack social context, or aim to replace bioethics by social science, or dismiss bioethics as a fig leaf for power altogether (Hoeyer 2006). These mainly sociological and anthropological studies focus on bioethics as their object, not bioethical problems and thus do not contribute to ethical theory-building. Being rather a critique of bioethics, they are only partly useful when trying to construct a critical bioethics.

However, other manifestations of the *social science critique* are more constructive, since they aim to redefine and enrich bioethics by methods and concepts from social sciences. The main problem to be solved by a critical approach in this understanding is the theory–practice divide (Hedgecoe 2004; Hoffmaster 1992). This social science critique opposes theory-laden bioethics, which often uses abstractions and speculations that have little to do with real-world problems (Mertz and Schildmann 2018). It fundamentally criticizes the focus on constructing rationally justified norms, principles, or theories and applying them to practice (Hoffmaster 1992). These constructs are mainly individualistic, i.e. focus on individuals as moral agents and universalistic, which means applicable in all cases (Callahan 1999). The prime example for this type of bioethics as applied ethics is principlism, which defines a set of prescriptive and binding principles and universalizes them. This variety of the *social science critique* claims that the applied-ethics understanding of bioethics ignores the theory–practice gap. By focusing on principles or theories as well as their application to practice, bioethics does not account for the social and cultural context in which decisions are made and actions are being taken (Hedgecoe 2004). Bioethical dilemmas are embedded in a social and cultural context and so are the moral systems bioethics is based on (Muller 1994). Hence, advocates of the social science critique argue that bioethics has to fully embrace social sciences, especially ethnography and anthropology, in terms of an empirical approach to ethical reasoning in real-world situations in order to bridge the theory–practice gap (Hedgecoe 2004; Muller 1994).

The empirical turn in bioethics (Borry et al. 2005) that occurred in the beginning of the century can be seen as a fulfillment of the social science critique. Empirical methods from the social sciences, mostly ethnographic methods like interview studies or participants observation, have been integrated into bioethics and are widely used in research projects nowadays (Mertz and Schildmann 2018).

Although the *analytical critique,* the *Socratic-hermeneutical critique,* and the *social science critique* each have their merit, they still fail in several ways. First, it has to be said that an analytical clarification of basic concepts is always crucial for every scientific endeavor. However, *analytical critique* alone is insufficient to make bioethical inquiry better suited for real-world problems, especially given the great social transformation described above. The same goes for the *Socratic-hermeneutical critique*, although it is at least aware of the social context in which bioethical thinking and its fundamental concepts are embedded. Both approaches, however, fail to address the power asymmetries as constituents of biomedical phenomena as well as bioethical inquiry. Awareness for conceptual clarity and social embeddedness aside, these two types of critique lack appropriate epistemic lenses and do not offer concrete methodological concepts on how to define and analyze the forces that shape bioethical reasoning and its objects.

Second, the *social science critique* understands the main task of bioethics in terms of clinical decision-making, thus reducing its spectrum to clinical ethics. It also supposes that applied ethics based on moral philosophy necessarily implies a top-down application from speculative theory to actual practice. It rightly states that traditional bioethics often does not reflect what counts as an ethical problem or ignores that what counts and what does not is a result of social construction. However, what the social science critique fails to see is that incorporating social factors is not limited to using empirical methods. It also requires the appropriate epistemic lenses for structuring, framing, and analyzing empirical data in order to uncover the societal power asymmetries that shape clinical medicine and biomedical research.

Normative ethics only makes sense if it is prescriptive, meaning that it defines the conditions of a morally right or wrong action. The true problem is not that bioethics is too theory-driven, on the contrary, it lacks the appropriate theoretical foundations in terms of epistemic lenses that allow to conceptualize bioethical problems as consequences of power asymmetries. Hence, critical bioethics in the full sense incorporates elements of the three types of criticism discussed above but adds epistemic lenses to make them work. These epistemic lenses already exist and were a product of a similar approach to understand and interpret fundamental sociopolitical changes in a way that allows for a new type of normativity. This approach is called critical theory.

## Why critical theory?

When speaking about critical theory, one can distinguish between two meanings. Critical Theory with capital letters usually refers to the so-called Frankfurt School, i.e. a philosophical and sociological school of thought that was established by members of the Institut für Sozialforschung (Institute for Social Research) in Frankfurt, Germany, at the beginning of the 1930s. Scholars like Theodor W. Adorno, Max Horkheimer and Herbert Marcuse introduced a Neo-Marxist approach that integrated such diverse elements as Neo-Hegelianism, Psychoanalysis, and Nietzschean thinking. The Frankfurt School contested neo-positivism as well as Heideggerian phenomenology, arguing that in their analyses contemporary philosophy and social science ignore the power-asymmetries inherent in capitalist societies. According to the Frankfurt School, theorizing about society is inherently normative, an emancipatory project, since it aims at uncovering power asymmetries that lead to social injustice. Any theorizing and also empirical research that ignores this fact is thus affirmative of those injustices. There have been several incarnations and interpretations of Critical Theory in different generations of the Frankfurt School following Horkheimer and Adorno, most notably Jürgen Habermas and Axel Honneth.

The other meaning is critical theory with lowercase letters, often also referred to as critical theories in plural. This is an umbrella term for approaches that embrace the concept of including power-asymmetries in philosophical and social science analysis. Feminist theories, critical race theory, postcolonialism, and some approaches from science and technology studies (STS) like critical data studies can be assigned to this spectrum. As diverse as these approaches are, what they have in common is the basic understanding that in contemporary societies, not all individuals are equal despite formal constitutional rights. These approaches address the power asymmetries in real-world social practices and social institutions in terms of political and social as well as epistemic inequality. A crucial aspect here is the link between epistemic and normative practices. How individuals or social groups, their traits, needs, and situation are framed shapes their social status as well as the social practices directed towards them.

Both types of critical theory can be an important source of concepts and epistemic lenses for critical bioethics. Regarding the lowercase variant, this has already been proven. There are strong currents of feminist bioethics as well as postcolonial approaches that contest mainstream bioethics. Interestingly, Critical Theory of the Frankfurt variant has not met with similar interest by bioethicists. I argue that this school of thought is an appropriate source for the epistemic lenses needed for contemporary bioethics.

One could argue that it would be easier to simply apply Critical Theory to bioethical issues instead of redefining bioethics. I think that there are two reasons against this. First, as a bioethicist, my aim is to contribute to the improvement of bioethics. I think that bioethical inquiry has proven its potential to support practitioners in the fields of healthcare and biomedical research as well as public health. Although a change of perspective is needed to better address current issues, bioethics is and should remain a field of inquiry in its own right. Second, there is a conceptual tension within critical theory when it comes to ethics in general and normative applied ethics in particular (Finlayson 2009; Honneth 2007; Keat 2008). Hence, one would first have to show that Critical Theory can be applied to ethical issues at all, which would demand an in-depth analysis beyond the scope of this paper. However, this question is of no importance to me since my focus is not on rethinking Critical Theory, but finding a new perspective for bioethics. Therefore, it suffices to take some concepts from Critical Theory and use them as epistemic lenses.

Why such an approach is needed becomes clearer when we look at the crucial challenges contemporary bioethics is facing. As stated above, these are mainly (1) inequity and inequality as a result of power asymmetries inherent to societies in late capitalism, (2) technological innovations, especially digital technologies, that disrupt social practices and relationships and imply a concentration of power and wealth in the hands of a few, (3) the political suppression of minority groups and all who disagree with techno-feudalist agenda. Critical theory offers epistemic lenses to address these issues, namely through its categories totality and embeddedness, instrumental reason, and emancipation (Celikates 2023; Rubeis 2024).

### Totality and embeddedness

Critical Theory emphasizes the sociohistorical context of theorizing and denies that theory is about finding some “suprasocial knowledge” (Horkheimer 1972). As Horkheimer states, the traditional understanding of theory seems to invoke a lone scholar detached from society reflecting on phenomena in an abstract manner. This ignores that the methods, concepts, and objectives of science are shaped by societal interests, be they a political agenda or of an economic nature. Hence, any theoretical approach has to reflect on the concrete social forces that shape it. What is more, it also has to consider how it contributes to the status-quo in society by simply reproducing concepts without questioning them and their social embeddedness. This is especially relevant regarding normativity in bioethics in the face of the sociopolitical shift we are experiencing. Take principlism for example. Instead of viewing autonomy and any other principles as existing in some abstract realm of ideas, one has to contextualize them with the concrete sociopolitical factors that shape them. This is essential in order to properly understand their intended meaning and also their conceptual limits.

The lens of totality and embeddedness is not limited to bioethical concepts or principles. As a materialist approach, Critical Theory is aware of the power asymmetries that exist in contemporary society and shape the experiences of individuals. This is the crucial trait that sets Critical Theory apart from other ways of analyzing social phenomena. It views individuals not as abstract or isolated agents that make decisions based on rational deliberation. Rather, it interprets them as being entangled in social practices and relationships that shape their perception as well as their agency. According to this view, individual needs and characteristics as well as the ability of individuals to make decisions and to act are mediated by social practices and processes (Adorno 1968). In order to understand and analyze these needs, characteristics etc. one has to consider the totality that shapes them. In Critical Theory, totality refers to society as the sum total of social practices, relationships, and the economic structure. In a bioethical context, this would imply to refute any analysis that views clinical decision-making as detached from the social totality and regards the patient-doctor dyad as an isolated encounter in some sort of a societal vacuum.

Critical bioethics thus rests on the fundamental concept of embeddedness that links individuals and their decisions as well as actions to the totality of societal power relations. This is a two-fold approach. First, actors in healthcare have to be regarded as embedded in different relationships and contexts. Their agency depends on their social status, age, gender, ethnicity, education, family status, and social determinants in general. This is obviously the case with patients, but also with healthcare professionals, since their role is defined by the medical community and comes with privileges, responsibilities, and expectations in terms of conduct. This undermines any atomistic understanding of autonomy that views actors as self-sufficient monads. This atomistic view rests mostly on a neoliberal understanding of the self and treats individuals as equal in bioethical inquiry although in many cases no real-world equality exists.

Second, the clinical encounter between doctor/nurse/therapist and patient/client is also embedded in a greater totality that shapes the roles as well as agencies of the actors involved and their relationship. This is especially relevant when it comes to decision-making. Other than the conventional bioethical understanding suggests, the crucial indicator of good decision-making is not only that it is based on information and a participatory approach. Rather than focusing solely on procedural fairness of the decision-making process, it has to be asked whether the available options are sufficient to enable autonomous and just decisions. For example, in a techno-feudalist setting, data practices might be arranged in a way to discriminate against certain minority groups by simply selecting specific training data or choosing varibales and parameters for data analysis in a way that excludes these groups. This lens helps to identify the factors that undermine an autonomous decision and a fair outcome beside the procedural rules of informed consent. In order to make this lens work, we have to make use of knowledge from disciplines that already incorporate power asymmetries in their analyses, like feminist, postcolonial, and other approaches.

### Instrumental reason

Critical Theory understands itself as a project of enlightenment, nevertheless reflects on the negative aspects of rationality, technology, and a positivist worldview. It recognizes the nexus between capitalism, science, and technology, in which technology cannot be detached from the business model behind it and regarded as a neutral tool (Marcuse 1964). Rationality always implies connecting ends with means in an efficient way. Instrumental reason specifically describes a type of rationality that focuses exclusively on ends-means-relations without questioning the ends (Horkheimer 2004). If the ends are not subject to normative deliberation and rational analysis only focuses on choosing the most efficient means for achieving a desired outcome, there is a certain risk of objectifying persons and relationships.

In medicine, such an instrumental reason is present in the technization of medical and care practices that goes hand in hand with their commodification. Technological solutions are often promoted as the ideal way of dealing with issues like lack of personnel or the demographic shift, two factors that increase the care burden. The discourse surrounding care robots may serve as an example here. Financial interests and political agendas are often the driving force in this context, responding to the care burden in a solutionist manner. That means that technology is seen as an instrument to resolve primarily social or political issues in an economically efficient way instead of addressing the underlying social and political issues that cause them. Technology might not be the adequate way of dealing with the issue at hand in terms of quality of care or well-being of healthcare professionals. It might simply be the most efficient way in an economic respect. Hence instrumental reason dictates to choose the most efficient means, which often simply means cost-efficient.

A critical bioethics would therefore not limit itself to weigh the benefits and risks of utilizing care robots against each other. It would use the epistemic lens of instrumental reason to analyze the reasons for investing in the development and utilization of robots instead of alternate solutions to the problem at hand, e.g. more and better trained personnel. This is just one example of many, especially regarding the massive endorsement of digital technologies for solving problems in healthcare we are witnessing at the moment. It would be crucial to use instrumental rationality as an epistemic lens for better understanding the ethical issues connected to the digital transformation of healthcare, especially when it comes to analyzing technology in connection with the business model behind it instead of a detached, neutral tool for solving problems.

### Dialectics and emancipatory thought

Dialectics might seem old-fashioned, something from the trash-heap of twentieth century Marxist or Neo-Hegelian philosophy. Nevertheless, Critical Theory shows how we can use dialectics to conduct normative analyses. Dialectics is basically uncovering contradictions between concepts like autonomy or justice and their enactment in real-life clinical situations. In Adorno’s words, “to call the spectre by its real name” (Adorno 1969) means to point where such a social practice is incongruent with the concept or principle it refers to. Hence, confronting a concept with the contradictions of its enactment is the necessary precondition for realizing the concept in full. Dialectics as a lens helps to identify and correct these contradictions instead of uncritically accepting a concept like autonomy as a description for the status quo.

Critical Theory is based on the assumption that an analysis of social phenomena is not a purely academic endeavor, but aims at social change and is thus normative. The basic ideas is to not take social phenomena as a given in a positivist manner, but to reflect on their normativity. Hence, Critical Theory confronts the objects of knowledge with their own concepts, in Adornos words (Adorno 1969). That means it identifies contradictions in society by pointing out the tension between what is and what is possible (Adorno 1969). To take another example, justice in medical practice does often not live up to its very concept due to the fact that power asymmetries exclude some individuals or social groups from epistemic practices. Minority groups are often under-represented in clinical studies, which shapes clinical epistemics and has normative consequences for clinical practice. As a consequence, their specific needs might be ignored. Hence, critique is the confrontation between a concept (like justice) and the object or practice it refers to (Adorno 1969). It does not treat such a concept as a given, reified category, but measures actual practice by how it lives up to the meaning of the concept. Furthermore, a critical approach does not simply uncover and condemn contradictions between a concept and its enactment in practice, but aims to show how this can be overcome (Müller-Doohm 2004). This enables a truly normative standpoint, one that does not only operate with accepted principles, but takes their normativity seriously by measuring clinical practice by this standard. Adorno’s negative dialectics in particular emphasizes the non-identity of the subject, meaning the refusal of totalizing tendencies within modernity that suppress individuality (Adorno 1990). Negative dialectics refutes any reconciliation of contradictions. Rather, it aims to make these contradictions visible in order to aim to emphasize and preserve the particularity of the subject.

Under the techno-feudalist paradigm, this emancipatory direction of bioethical thought becomes even more important. In addition to structural discrimination that already exists, new and more direct forms of discrimination are to be expected. This has already become a reality in the USA where the NIH is not allowed to use specific words like “diversity” or “woman/female” in official documents or fund projects that aim at health equity for minority groups (van Daal et al. 2025). Given these openly discriminatory practices, bioethics cannot contend itself with weighing principles against each other. Rather, bioethics has to speak out against this type of suppression and social injustice and not just by publishing viewpoint articles and the like, but by integrating the emancipatory stance based on dialectic thinking into bioethical theory-building and inquiry. This emancipatory normativity is the very essence of critical bioethics and can also be seen as a possible link between bioethics and other emancipatory approaches, such as feminist ethics, queer theory, animal rights or deep ecology. It would be particularly interesting to make this connection in further research. For example, integrating a deep ecology perspective in a critical bioethics inquiry could help to bridge the ecosystemic gap between medicine and ecology (Boudreau LeBlanc 2023), thus providing a more holistic perspective and enabling emancipatory action.

## Conclusion

By using crucial concepts from critical theory as epistemic lenses, critical bioethics is suited to deal with the contemporary challenges mentioned above: It addresses inequity and inequality as a result of power asymmetries, viewing individuals as fundamentally embedded in a concrete socioeconomic context. It reflects upon disruptive technological innovations and the connection between technological solutionism and commodification through the lens of instrumental reason. It provides an emancipatory perspective for normativity by using dialectics to uncover contradictions in regard to bioethical principles.

This paper is an attempt to outline the general direction and perspective of critical bioethics. It argues for a paradigm shift in bioethics in order to be able to face the challenges resulting from the social disruption we are witnessing in Western societies. However, the real work starts where this paper ends. Once this paradigm has been established and the epistemic lenses have been introduced, future research can use this for concrete analyses of particular issues. This may also include a deeper dive into Critical Theory, since further categories could be useful as epistemic lenses in bioethics. Furthermore, it would be interesting to engage further with critical theories (feminist theory, postcolonial thought, critical race theory, etc.) to unlock their potential for critical theorizing in bioethics.
